# Mechanical and dynamic characterization of prosthetic feet for high activity users during weighted and unweighted walking

**DOI:** 10.1371/journal.pone.0202884

**Published:** 2018-09-12

**Authors:** Sara R. Koehler-McNicholas, Eric A. Nickel, Kyle Barrons, Kathryn E. Blaharski, Clifford A. Dellamano, Samuel F. Ray, Barri L. Schnall, Brad D. Hendershot, Andrew H. Hansen

**Affiliations:** 1 Minneapolis Department of Veterans Affairs Health Care System, Minneapolis, Minnesota, United States of America; 2 Department of Biomedical Engineering, College of Engineering, University of Minnesota, Minneapolis, Minnesota, United States of America; 3 Department of Rehabilitation, Walter Reed National Military Medical Center, Bethesda, Maryland, United States of America; 4 Department of Defense-Veterans Affairs Extremity Trauma and Amputation Center of Excellence, Bethesda, Maryland, United States of America; 5 Department of Rehabilitation Medicine, Uniformed Services University of the Health Sciences, Bethesda, Maryland, United States of America; 6 Program in Rehabilitation Science, Department of Rehabilitation Medicine, University of Minnesota, Minneapolis, Minnesota, United States of America; University of Colorado Boulder, UNITED STATES

## Abstract

Many Service members and Veterans with lower-limb amputations have the potential for high function and the desire to resume physically demanding occupations that require them to carry heavy loads (e.g., military service, firefighters, farmers, ranchers, construction workers). However, it is currently unclear which prosthetic feet best accommodate heavy load carriage while also providing good overall function and mobility during unweighted activities. The main objective of this study was to investigate the ability of currently available prosthetic ankle-foot systems to accommodate weighted walking by examining the mechanical characteristics (i.e., forefoot stiffness) and dynamic function (i.e., rocker radius, effective foot length ratio, and late-stance energy return) of prosthetic feet designed for high activity users. Load versus deflection curves were obtained for nine prosthetic ankle-foot systems using a servohydraulic test frame and load cell. Effective roll-over shape characteristics and late-stance energy return measures were then obtained using quantitative gait analysis for three users with unilateral, transtibial amputation. Results from mechanical and dynamic testing showed that although forefoot stiffness varied across the nine feet investigated in this study, changes measured in roll-over shape radius and effective foot length ratio were relatively small in response to weighted walking. At the same time, prosthetic feet with more compliant forefoot keel structures appeared to provide more late-stance energy return compared to feet with stiffer forefoot keel structures. These results suggest that prosthetic ankle-foot systems with compliant forefoot keel structures may better accommodate weighted walking by reducing the metabolic cost of physically demanding activities. However, to more fully understand the biomechanical and functional implications of these results, other factors, such as the residual-limb strength of the user and the overall stiffness profile of the prosthetic foot, should also be considered.

## Introduction

The ability to wear or carry loads beyond body weight is important for functional independence and is required in many occupational contexts (e.g., military service, firefighters, farmers, ranchers, construction workers). With regard to the military, the mass of protective gear and weapons/ammunition alone can be upwards of 21 kg (46 lb) and light infantry troops often carry loads of 45 kg (100 lb) or more during dismounted operations [1]. While numerous studies have evaluated the biomechanical and energetic consequences of load carriage in able-bodied individuals (e.g., [2–4]), only a few have investigated the comparable effects of load carriage in Service members with combat-related amputations, many of whom wish to return to active duty or other highly active/demanding occupations. Schnall et al. [5] found that compared to able-bodied individuals, lower-limb prosthesis users exhibit greater metabolic costs while walking with added loads, both at mid-range and high-end speeds of military foot marches. Other studies have shown that during weighted walking, lower-limb prosthesis users exhibit greater (and asymmetrical) demands on the musculoskeletal system [6] and larger deflections of the prosthetic ankle-foot system compared to unweighted walking [7,8]. The latter, in particular, suggests additional work focused specifically on the functional implications of these load responses is warranted.

Clinicians treating Service members and Veterans with a lower-limb amputation have a wide variety of prosthetic components to choose from; yet there remains a general lack of objective criteria for evaluating and prescribing prosthetic ankle-foot components [9,10], and none specifically for load carriage activities. During weighted walking, individuals with a healthy, intact ankle-foot complex maintain similar ankle joint kinematics and ankle-foot roll-over shapes [11,12], suggesting substantial internal joint moments are generated to counter externally applied forces and moments and effectively vary joint stiffness. Most current prosthetic ankle-foot systems, however, are not capable of providing such variations in joint stiffness in response to changing external demands. Instead, most prosthetic feet deflect proportionally with added loads, thereby resulting in increased prosthetic ankle dorsiflexion [7,8] and presumably, decreased roll-over shape radii compared to unweighted walking. To counteract this deflection, recent experimental studies have shown that increasing prosthetic forefoot stiffness can significantly decrease ankle dorsiflexion [13,14]. However, prosthetic feet with stiffer forefoot keel structures have also been shown to provide less late-stance energy return [14], highlighting a potential trade-off in the prescription strategy of feet for highly active users.

**Fig 1:**
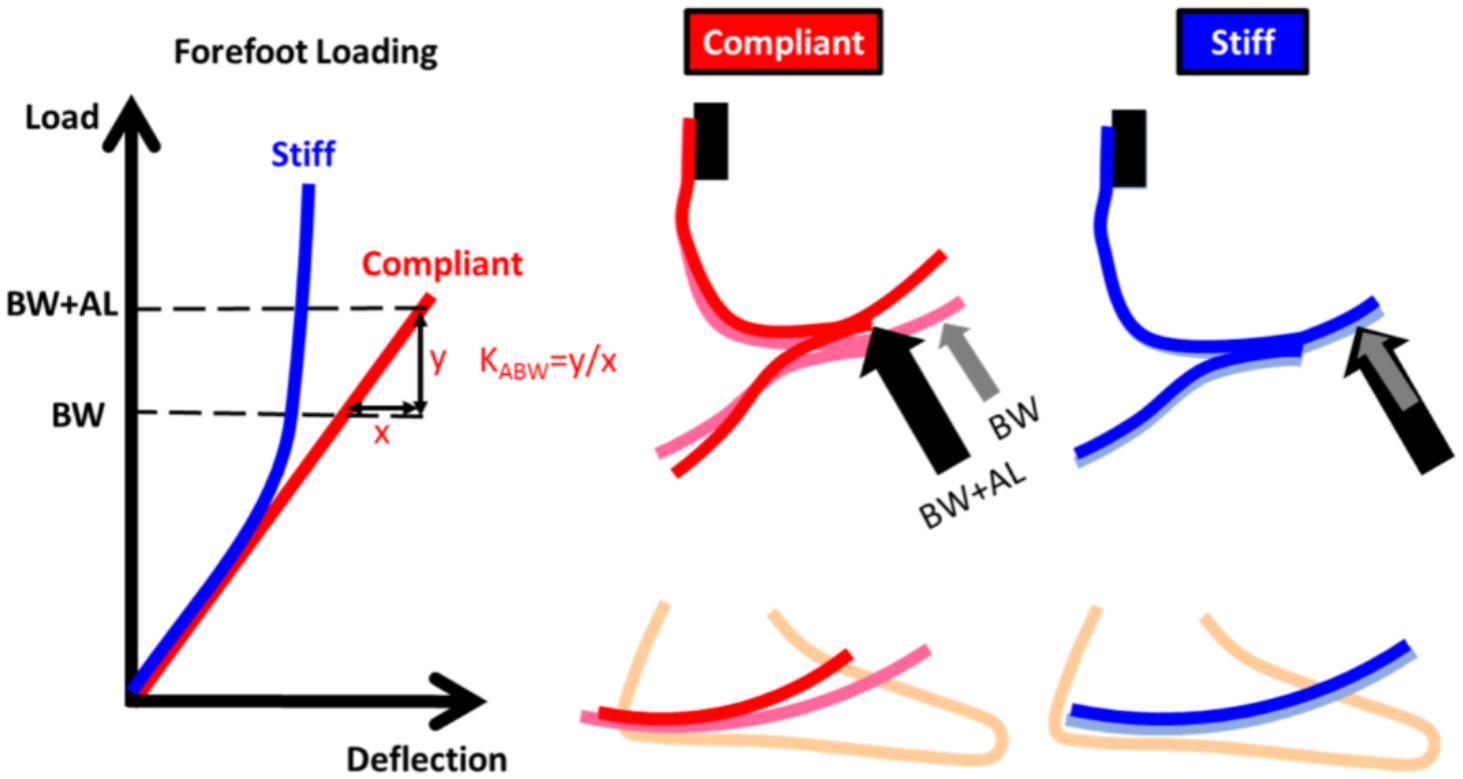
Prosthetic feet with stiff forefoot keel structures should conform to more consistent roll-over shapes when walking with added loads compared to feet with compliant forefoot keel structures. Compliant prosthetic feet (red, middle) will continue to bend when users carry their body weight (BW) plus added loads (AL). This continued bending should lead to a roll-over shape with a smaller radius. Stiff prosthetic feet (blue, right) should have only a slight amount of additional bending when users carry added loads. K_ABW_ = forefoot stiffness at loads above body weight.

Accordingly, the main objective of this study was to investigate the ability of currently available prosthetic ankle-foot systems to accommodate weighted walking by examining the mechanical characteristics (i.e., forefoot stiffness) and dynamic function (i.e., rocker radius, effective foot length ratio, late-stance energy return) of prosthetic feet designed for the highest activity users. In order to evaluate forefoot stiffness, load versus deflection curves were obtained for nine different prosthetic ankle-foot systems using a servohydraulic test frame and load cell. Following mechanical testing, three research participants were recruited to walk with each prosthetic ankle-foot system and quantitative gait analysis was used to obtain effective roll-over shape and energy return data. We hypothesized that for prosthetic ankle-foot systems with compliant forefoot keel structures, added loads would be associated with larger deformations of the prosthetic forefoot, thereby reducing roll-over shape radii compared to unweighted walking (Fig 1, red). In contrast, we hypothesized that prosthetic ankle-foot systems with stiff forefoot keel structures would better accommodate weighted walking, as evidenced by smaller changes in roll-over shape radii with added load (Fig 1, blue). We further hypothesized that ankle-foot systems with compliant forefoot keel structures would provide more late-stance energy return compared to systems with stiff forefoot keel structures. Collectively, we expect the results of this study to be useful in guiding the selection of prosthetic feet for Service members who want to return to active duty and for individuals with lower-limb amputation who want to engage in activities that require carrying added loads.

## Methods

### Selection of ankle-foot prostheses

Nine prosthetic ankle-foot systems, all marketed for users in the highest Medicare Functional Classification Level (MFCL K4), were investigated in this study: 1) Renegade AT (Freedom Innovations, Irvine, CA), 2) Thrive (Freedom Innovations, Irvine, CA), 3) Variflex XC (Össur, Reykjavik, Iceland), 4) Soleus Tactical (College Park, Warren, MI), 5) Triton Heavy Duty (Otto Bock, Duderstadt, Germany), 6) All Pro (Fillauer, Chattanooga, TN), 7) Rush Foot (Ability Dynamics, Tempe, AZ), 8) Trekk (Makstride Prosthetics, Prescott, AZ), and 9) Panthera CFII (mediUSA, Whitsett, NC). Prosthesis selection was based on several factors important for Service members returning to active duty: 1) no moving parts, which generally require less maintenance and are less prone to failure over time, 2) feet with long, spring-like keel structures, which should experience less strain for a given deflection and therefore be more robust, and 3) feet with thick keel structures, which are recommended for high impact activities required in active duty. Despite the potential for all nine feet to be appropriate for MFCL K4 users, distinguishing features in their mechanical design (e.g., the Thrive’s dual-keel configuration) suggested that some feet may exhibit more consistent roll-over shapes when walking with added loads than others. Three units were purchased for each ankle-foot type based on the body weight and foot length of three users identified for human subject testing. Accordingly, twenty-seven ankle-foot prostheses were characterized during mechanical and human subject testing.

### Mechanical characterization of ankle-foot prostheses

**Fig 2:**
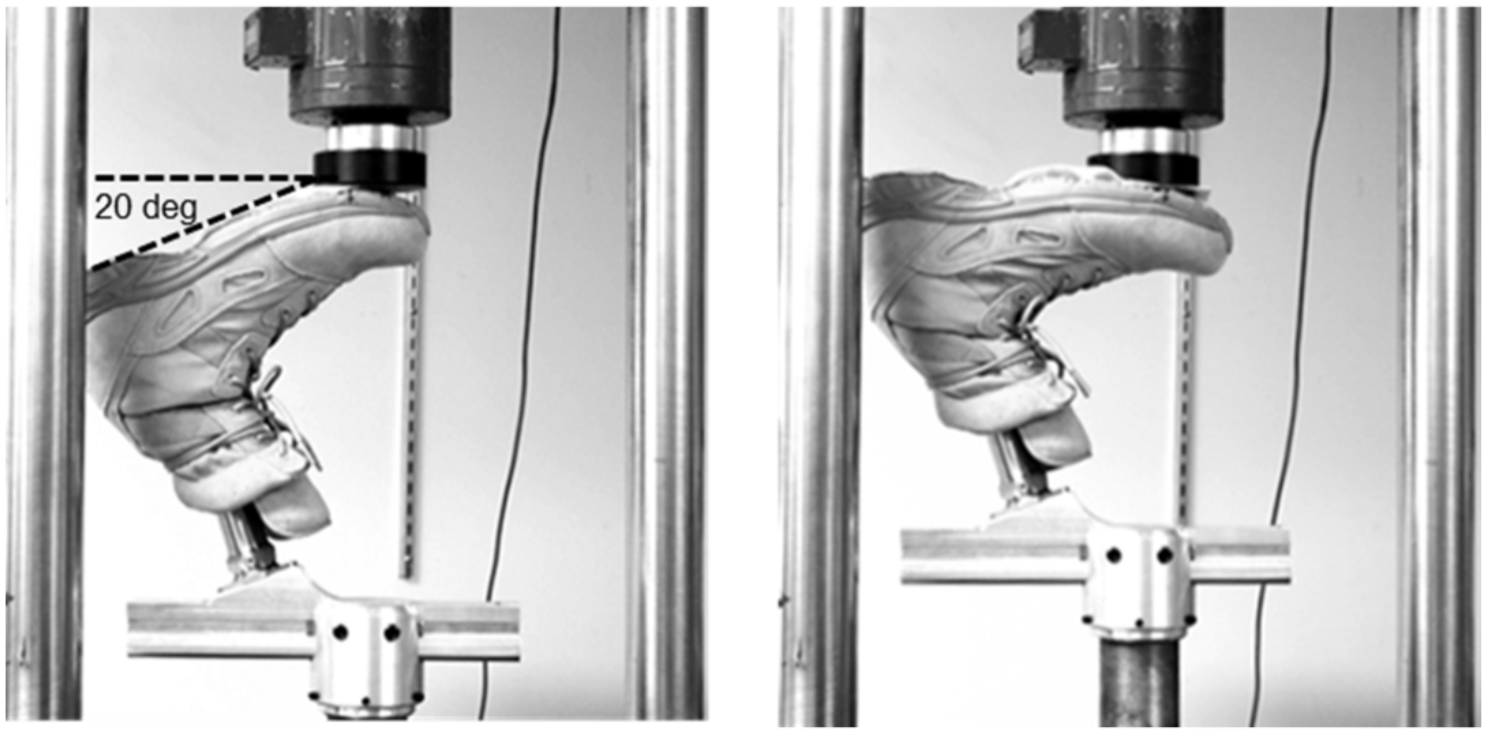
Load versus deflection profiles for each foot were obtained using a servohydraulic universal test frame (MTS 858, MTS, Eden Prairie, MN) with axial/torsional capabilities, a computer-based data acquisition system (Wintest, Bose, Framingham, MA), and a load cell (MTS 661-21A-01, MTS, Eden Prairie, MN). The load frame applied uniaxial loads to the foot.

Prior to human subject testing, load versus deflection profiles for each foot were obtained using a servohydraulic universal test frame (MTS 858, MTS, Eden Prairie, MN) with axial/torsional capabilities, a computer-based data acquisition system (Wintest, Bose, Framingham, MA), and a load cell (MTS 661-21A-01, MTS, Eden Prairie, MN). The load frame applied a uniaxial load to the foot as shown in Fig 2. The load frame recorded deflection, while the load cell recorded applied load.

To approximate conditions expected during active duty military activities, each foot was tested in a “use state” (i.e., placed within a foot shell and a Reebok Men's Hyper Velocity 8-inch UltraLight Performance boot). A sheet of adhesive-backed polytetrafluoroethylene (PTFE) was also placed on the tread of the boot to provide a low friction interface with the loading platform. All feet were mounted in a fixture such that the plantar surface of the boot was set at a 20-degree angle from horizontal, simulating forefoot loading as defined by the ISO 10328 standard (Fig 2). Different foot components were aligned in the load frame with a mark on the boot near the “ball” of the foot. Prior to loading, the 20-degree angle was verified using a digital inclinometer placed on the non-deformed sole of the boot.

Each foot was loaded to body weight + vest weight (22 kg), approximating loading conditions associated with the second peak of the vertical ground reaction force of transtibial prosthesis users walking at speeds between 1.2–1.6 m/s [15]. Test loads were applied at a rate of 100 N/s, held for 1s, then ramped down at a rate of 100 N/s and held at 0 N for 1s. This cycle was applied nine times for each foot. Each foot was visually inspected after the test cycle to ascertain whether there was any evidence of breakage or failure.

To compare the mechanical properties of each foot, load versus deflection profiles from the ninth loading cycle were plotted from 50 N of applied load to the maximum load. The ninth loading cycle was used for analysis to allow the foot to settle into a consistent position on the load frame. The best-fit linear slope of the load versus deflection curve was then calculated between body weight and 22 kg above body weight for each respective subject, corresponding to forefoot stiffness at loads above body weight (K_ABW_). Stiffness values were normalized by body weight in order to calculate means across subjects.

### Dynamic characterization of ankle-foot prostheses

Human subjects testing was approved by the Minneapolis VA Health Care System’s Institutional Review Board: 4523-B Characterization of Prosthetic Feet for Weighted Walking in Service Members with Lower-Limb Amputation. Data were collected on three subjects with unilateral, transtibial amputation who provided their informed written consent. Subjects were recruited based on the following inclusion criteria: Veterans between the ages of 18–50 years, transtibial amputation with non-vascular etiology, MFCL K4 as determined by a clinical prosthetist, endoskeletal prosthesis with enough clearance to test the ankle-foot systems under investigation, able to understand informed consent, and six or more months of experience with a definitive prosthesis. Subjects were excluded from the study if they presented with a sore on their residual limb, had a health condition that contraindicated participation in a weighted walking study, or had a poorly fitting socket.

**Fig 3:**
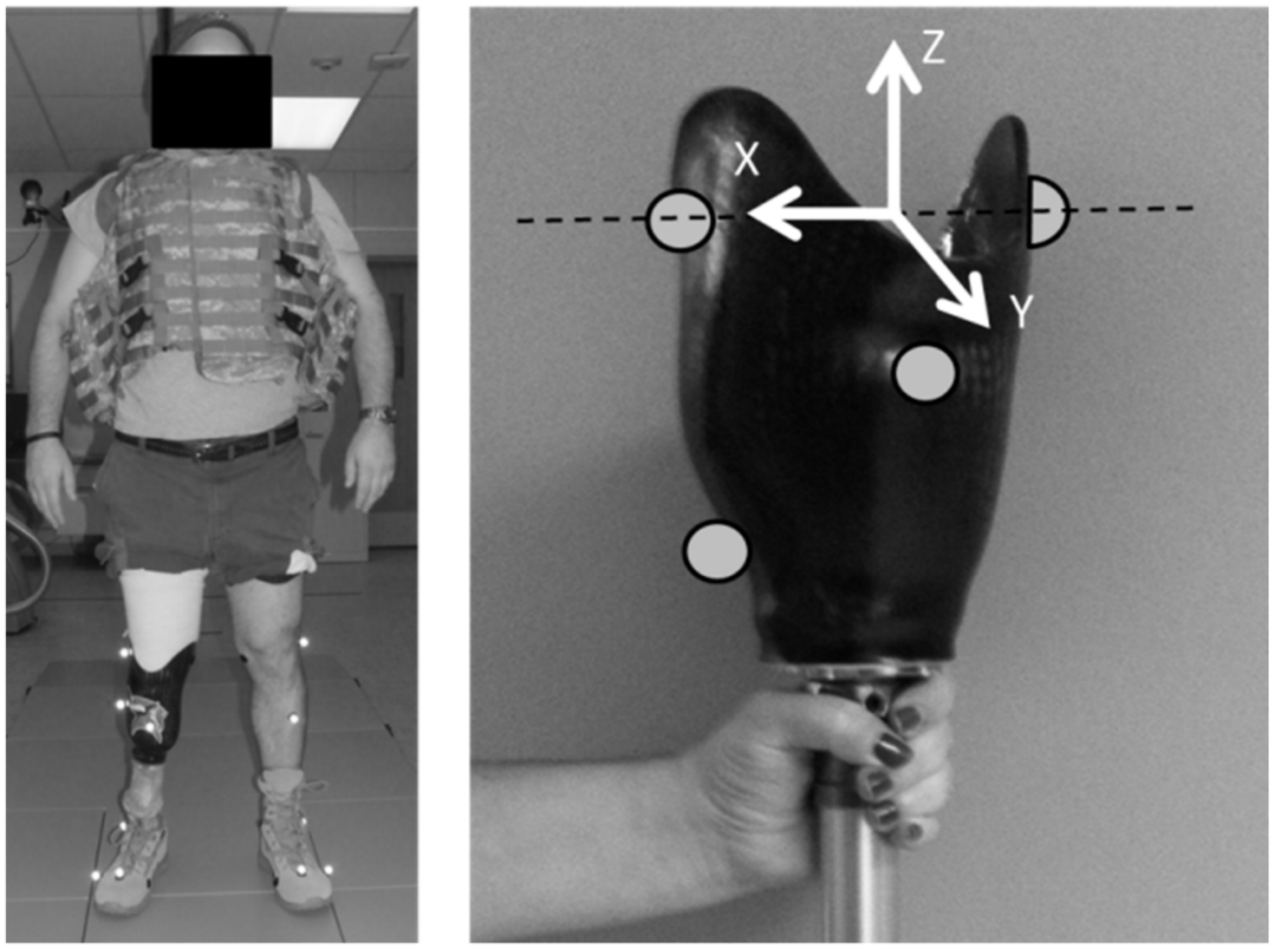
Marker placement for roll-over shape characterization. Subjects wore a 22-kg vest for all weighted walking conditions (left). Center of pressure data were transformed into an anatomically relevant socket coordinate system (right) in order to calculate roll-over shapes for each prosthetic ankle-foot system under investigation.

Each subject was involved in the study for one visit, during which time a clinical prosthetist disconnected the subject’s residual-limb socket from the rest of their prosthesis using a procedure that preserved the prosthetic alignment of their usual prosthesis for re-attachment at the end of the study [16]. The prosthetist then fit the first prosthetic foot to the subject’s residual-limb socket using standard clinical procedures. The military boot used during mechanical characterization was also used during human subject testing. After clinical optimization of the alignment, the subject walked for several minutes over level ground to become accustomed to the foot design. Following this accommodation period, reflective markers were placed on their residual-limb socket to define an anatomically relevant socket coordinate system (Fig 3, right [17]).

Subjects then walked without added weight across two AccuGait force platforms (AMTI, Watertown, MA; sampling rate = 1200 Hz) mounted flush within a surrounding 3.4 m walkway while an 8-camera Oqus 100 motion analysis system (Qualisys Motion Capture Systems, Gothenburg, Sweden; sampling rate = 120 Hz) tracked the reflective markers on their socket. Subjects walked at their normal speed until at least five clean force platform hits had been collected. During this first condition, the subject’s walking speed was recorded; during all subsequent trials, walking speed was monitored to ensure that the subject maintained a comparable speed. Subjects were then fitted with a weighted (22 kg) vest (Point Blank Enterprises, Inc., Pompano Beach, FL; Fig 3, left) that simulated the fighting load of Service members currently engaged in combat (i.e., ballistic protective vest with load bearing equipment) and repeated the testing protocol. Once finished, the vest was removed and the prosthetist fit and aligned the next prosthetic foot. Prosthetic feet were tested in random order until subjects had walked with all nine feet.

### Data analysis

Raw marker data were processed using Qualisys Track Manager (QTM 2.11), then exported into MATLAB^®^ (R2010b, Mathworks, Inc., Natick, MA) for further analysis. To calculate the roll-over shape of each prosthetic foot, the center of pressure of the ground reaction force was transformed into a socket-based coordinate system with its origin at the knee center [17]. Given the inherent uncertainty of center of pressure data at low force levels, a force threshold of 150 N was applied to ground reaction force data and roll-over shapes were calculated during the single-support phase of gait (i.e., between contralateral toe off and heel strike). The best-fit circular arc for each roll-over shape was then calculated in order to determine the mean roll-over shape radius (normalized by height) for each foot [12]. To quantify the effect of added weight on roll-over shape radius, within-subject differences were calculated between the mean unweighted and weighted radii for each foot, then averaged across subjects.

From roll-over shape data, the effective foot length ratio of each foot was also calculated according to methods described previously [18]. This measure represents the fraction of the total foot length that is effectively used during the single-support phase of gait. Similar to the roll-over shape analysis, within-subject differences between the mean unweighted and weighted effective foot length ratios were calculated for each foot and averaged across subjects.

Finally, a unified deformable (UD) segment analysis [19] was used to calculate total energy return of the keel using Visual3D (C-Motion, Inc., Germantown, MD). Compared to a traditional inverse dynamics analysis, the UD segment analysis considers all components below a rigid prosthetic socket a deformable mass to more accurately capture the energetics of prosthetic structures. In this study, the proximal rigid segment was defined and tracked using markers on the residual-limb socket (Fig 3, right). Markers placed on the lower limb were used to calculate shank center of mass according to Visual 3D’s built-in estimator, which uses able-bodied anthropomorphic tables to calculate segment mass and moment of inertia. Total energy return of the keel was then quantified by integrating all power done by the prosthesis between zero crossings near the end of single support phase and at toe off. These data were averaged for each foot and weight condition across their respective trials and normalized by subject mass (including the 22-kg mass when relevant) to determine the mean energy return values for each prosthesis in both the weighted and unweighted conditions.

## Results

### Subject demographics

Data were collected from three male subjects with unilateral, transtibial amputation. Amputation etiology included trauma (2 subjects) and bone lesion (1 subject). The mean age, mass, and height of the subject population was 39 ± 6 years, 85 ± 14 kg, and 1.76 ± 0.05 m, respectively (Table 1). All subjects had at least 31 months of experience with a definitive prosthesis (Table 1). The self-selected walking speed of the group ranged from 1.23–1.49 m/s. Once selected, subjects maintained a similar walking speed across all test conditions (Table 1).

**Table 1:** Subject demographics.

Subject	Age (years)	Body mass (kg)	Height (m)	Foot Length (cm)	Months since amputation	Months with definitive prosthesis	Walking speed (m/s)
**A**	44	70	1.78	27	33	31	1.46 ± 0.02
**B**	32	89	1.70	25	40	36	1.23 ± 0.02
**C**	42	97	1.80	27	37	35	1.49 ± 0.02

Walking speed represents mean speed across all test conditions (± 1 standard deviation).

### Mechanical characterization of ankle-foot prostheses

**Fig 4:**
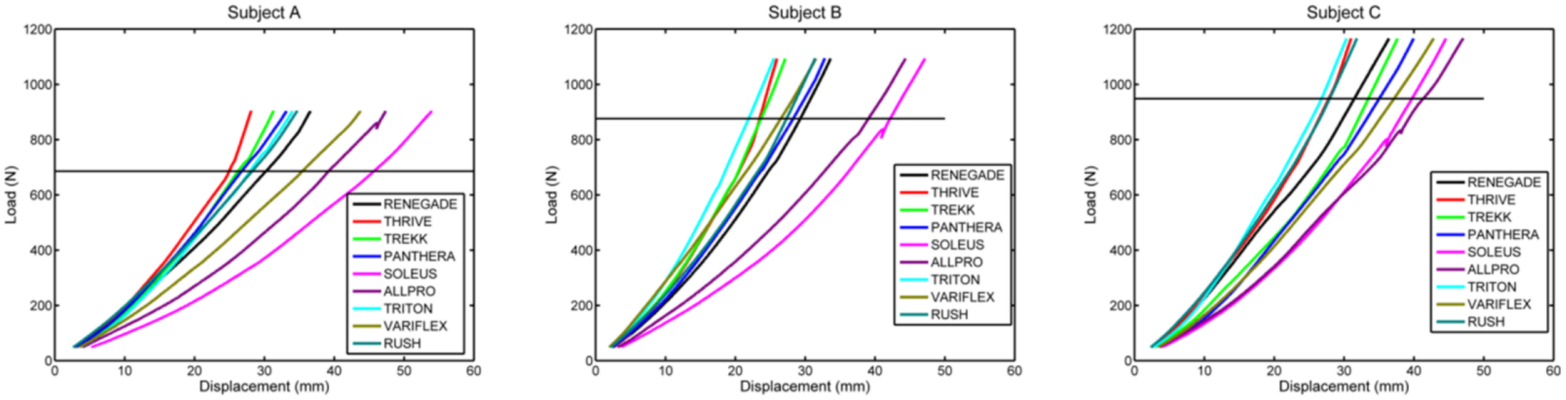
Load versus displacement curves from the ninth loading cycle obtained during mechanical characterization. The horizontal line represents body weight. Maximum load represents body weight plus the weighted vest (22 kg). Small discontinuities evident in some curves (e.g., near the maximum load of Subject A using the Soleus) represent momentary sticking at the loading interface despite the low-friction PTFE sheet affixed to the boot tread.

Fig 4 shows the results of mechanical testing, with load versus deflection curves grouped according to test subject. Although stiffness profiles varied across prosthetic feet, it is interesting to note that for all three subjects, the Soleus and All Pro consistently appeared to be the least stiff (i.e., most displacement per unit load) and the Thrive and Triton appeared to be the most stiff.

**Fig 5:**
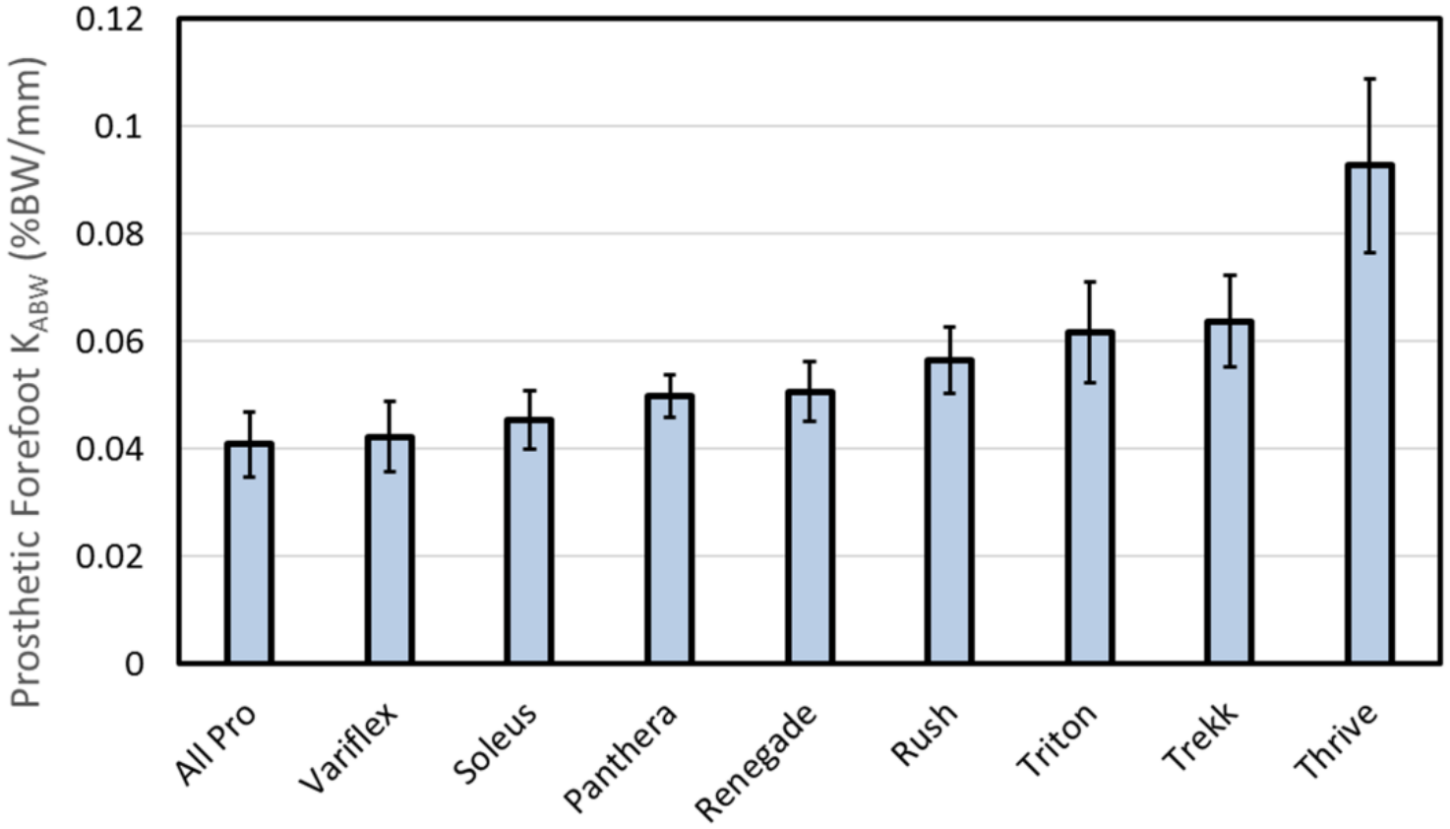
Mean (± 1 standard deviation) forefoot stiffness at loads above body weight (K_ABW_). Results are sorted from least (left) to greatest (right) forefoot stiffness.

Mean calculated forefoot stiffness is quantified in Fig 5. Stiffness values are sorted from lowest (All Pro = 0.04 ± 0.006%BW/mm) to highest (Thrive = 0.09 ± 0.02%BW/mm). The mean coefficient of determination (r^2^) of the best-fit linear slope of the load versus displacement curve above body weight was 0.996 ± 0.006 (range: 0.969–1.000) across all feet.

### Roll-over shape characterization of ankle-foot prostheses

**Fig 6:**
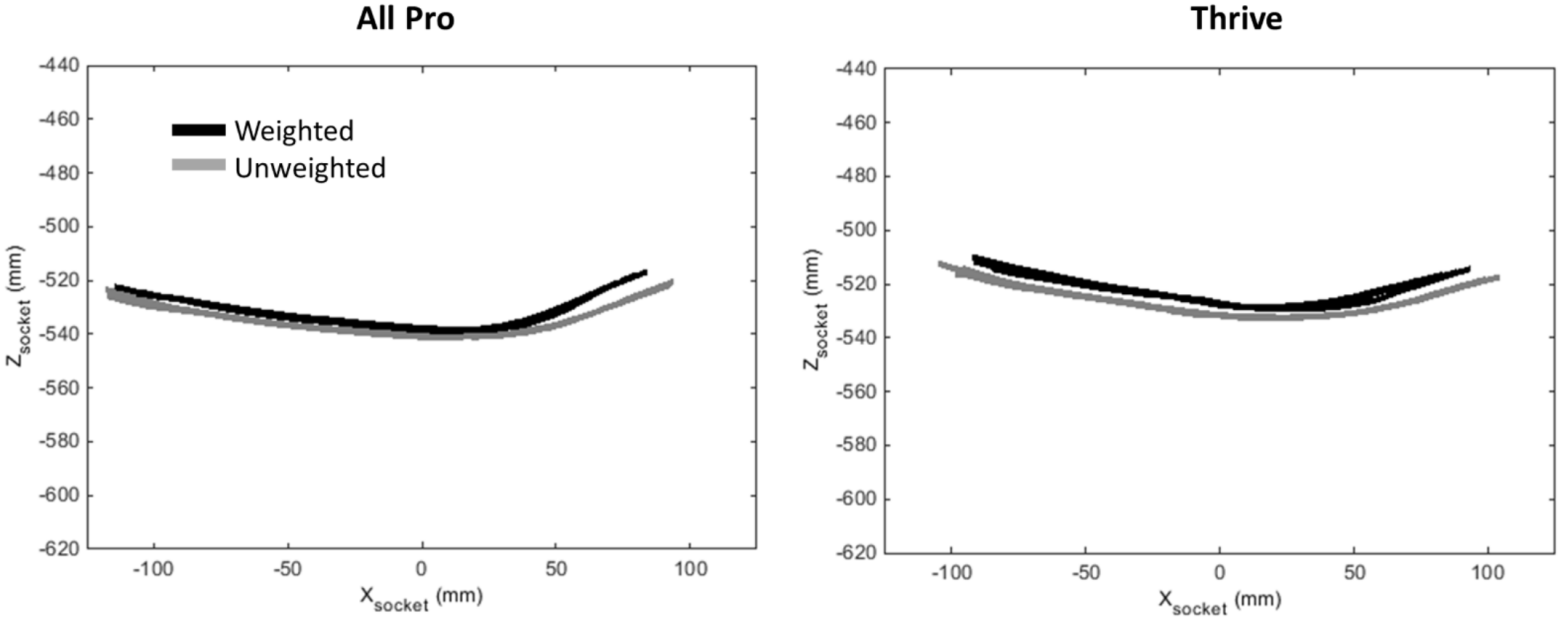
Roll-over shapes from all trials of the All Pro and Thrive during weighted and unweighted walking for Subject A. Roll-over shapes are shown in a socket-based coordinate system with the origin at the knee center.

**Fig 7:**
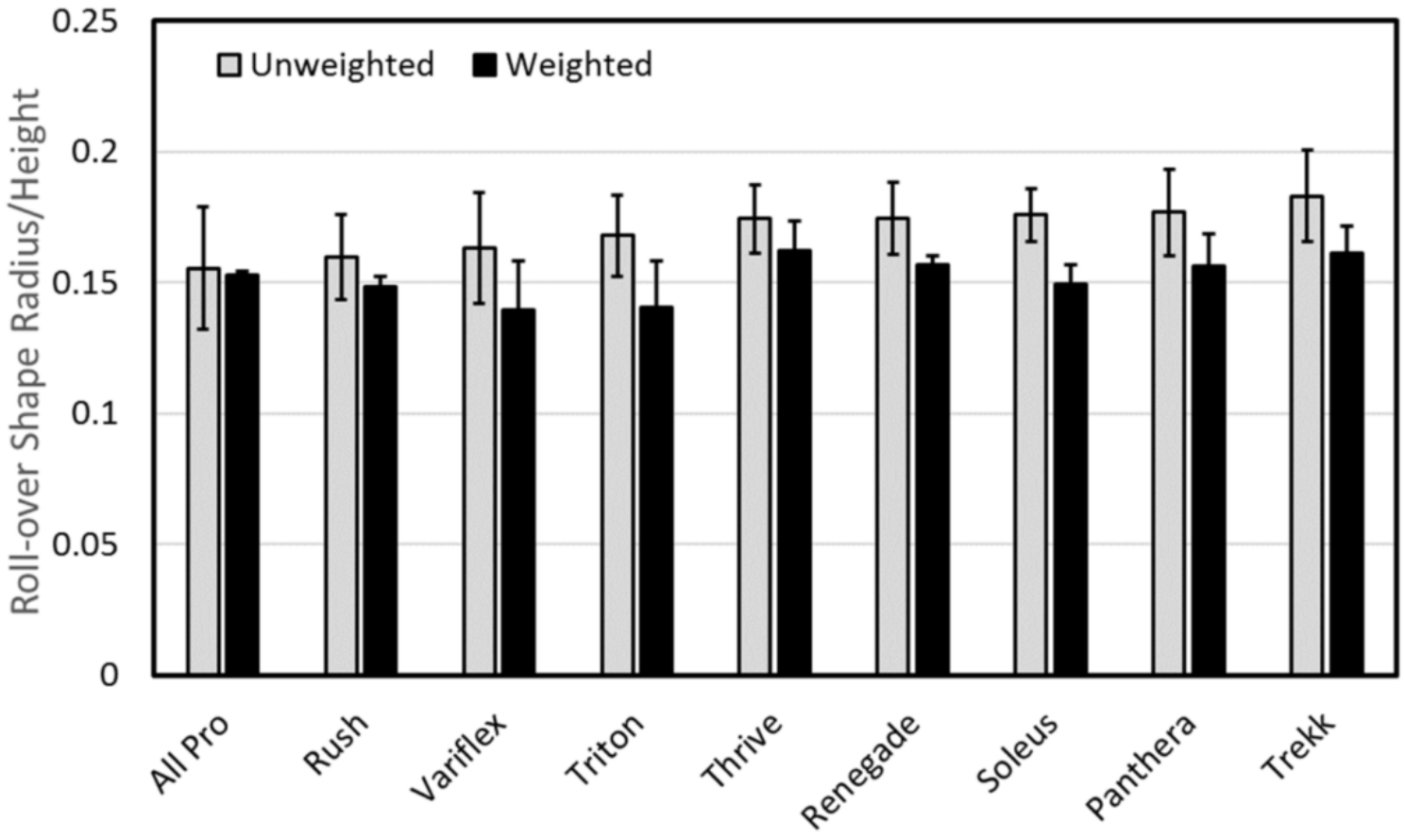
Mean (± 1 standard deviation) roll-over shape radii (normalized by height) across all subjects. Results are sorted from smallest (left) to largest (right) mean unweighted roll-over shape radius for each foot.

Despite differences observed in forefoot stiffness during mechanical testing, roll-over shape profiles measured during human subject testing appeared relatively similar between the weighted and unweighted walking conditions for all 27 ankle-foot prostheses tested in this study. Fig 6 shows all trials of Subject A wearing the All Pro (i.e., least stiff) and Thrive (i.e., most stiff) during both the weighted and unweighted walking conditions. Fig 7 shows the mean roll-over shape radius across all subjects for each foot. For the unweighted walking condition, the mean roll-over shape radius (normalized by height) across feet was 0.170 ± 0.009 and ranged from 0.156 ± 0.023 for the All Pro to 0.183 ± 0.017 for the Trekk. For the weighted walking condition, the mean roll-over shape radius across feet appeared to decrease to 0.152 ± 0.008 and ranged from 0.140 ± 0.019 for the Variflex to 0.162 ± 0.012 for the Thrive.

**Fig 8:**
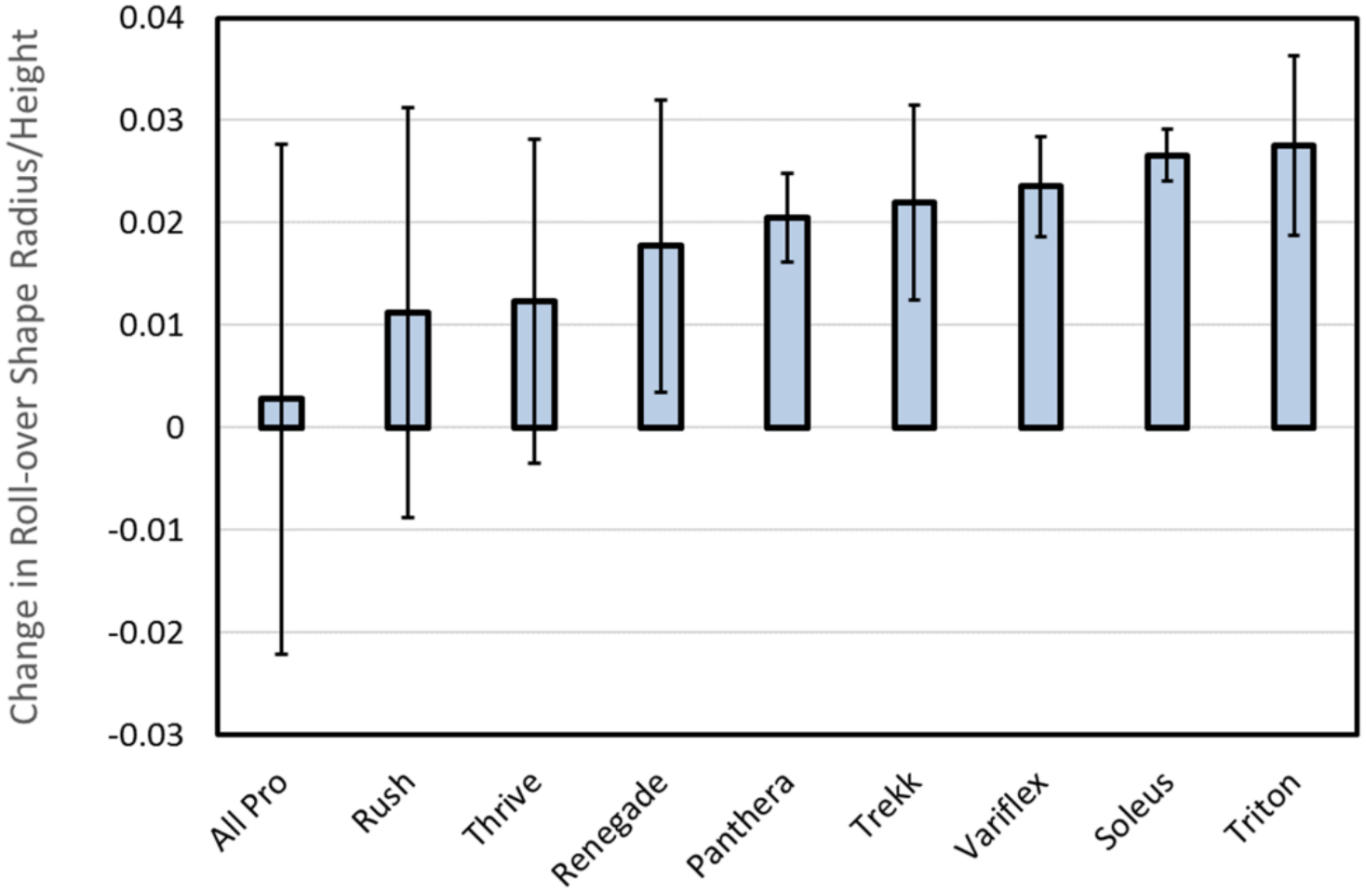
Mean (± 1 standard deviation) change in roll-over shape radius (unweighted—weighted) across all subjects. Results are sorted from the smallest (left) to largest (right) difference in roll-over shape radii (normalized by height).

Fig 8 shows the mean change in roll-over shape radius due to added weight across all subjects, sorted from the smallest difference (All Pro = 0.003± 0.025) to the largest difference (Triton = 0.028 ± 0.009). Standard deviation bars for the All Pro, Rush, and Thrive feet are larger than the other six feet due to the fact that Subject B exhibited a smaller roll-over shape radius for the unweighted versus the weighted condition. For all other ankle-foot systems analyzed in this study (i.e., 24 out of 27 feet), roll-over shape radius consistently decreased during weighted versus unweighted walking.

### Effective foot length ratio of ankle-foot prostheses

**Fig 9:**
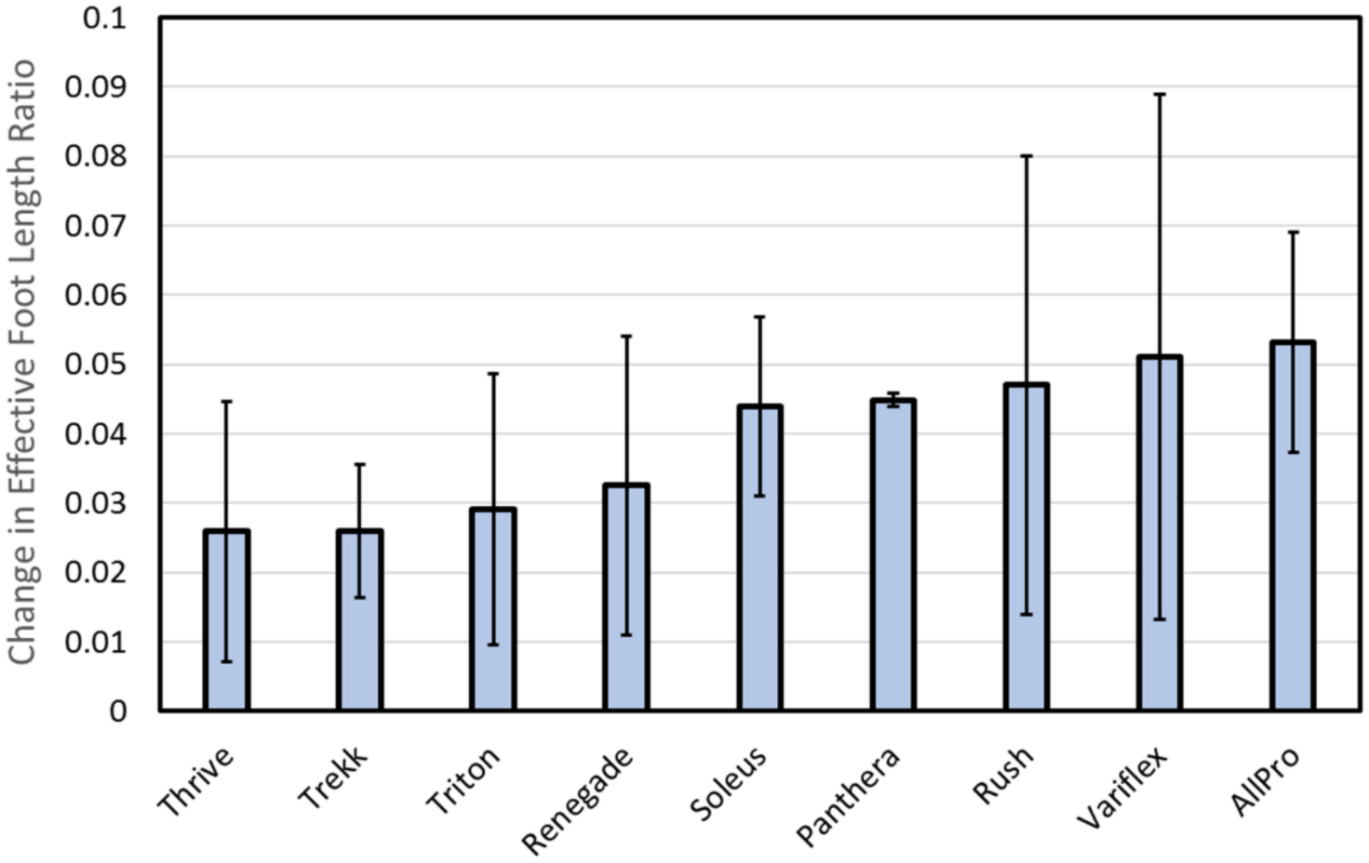
Mean (± 1 standard deviation) change in effective foot length ratio (unweighted—weighted) across all subjects. The effective foot length ratio is a fraction of the total foot length that is effectively used during the single-support phase of gait. Results are sorted from the smallest (left) to largest (right) difference in effective foot length ratio.

Fig 9 shows the effect of added weight on changes in effective foot length ratio calculated across all subjects. As shown in this figure, mean effective foot length ratio changed less than 0.053 across all feet. Data shown in Fig 9 have been sorted from the foot with the smallest change in effective foot length ratio (Thrive = 0.026 ± 0.019) to the foot with the largest change in effective foot length ratio (All Pro = 0.053 ± 0.016 mm). Feet with the lowest forefoot stiffness measured during mechanical testing (All Pro and Variflex) appeared to exhibit the largest change in effective foot length ratio due to added weight, whereas feet with the highest forefoot stiffness (Trekk and Thrive) appeared to exhibit the smallest change in effective foot length ratio due to added weight.

### Late-stance energy return of ankle-foot prostheses

**Fig 10:**
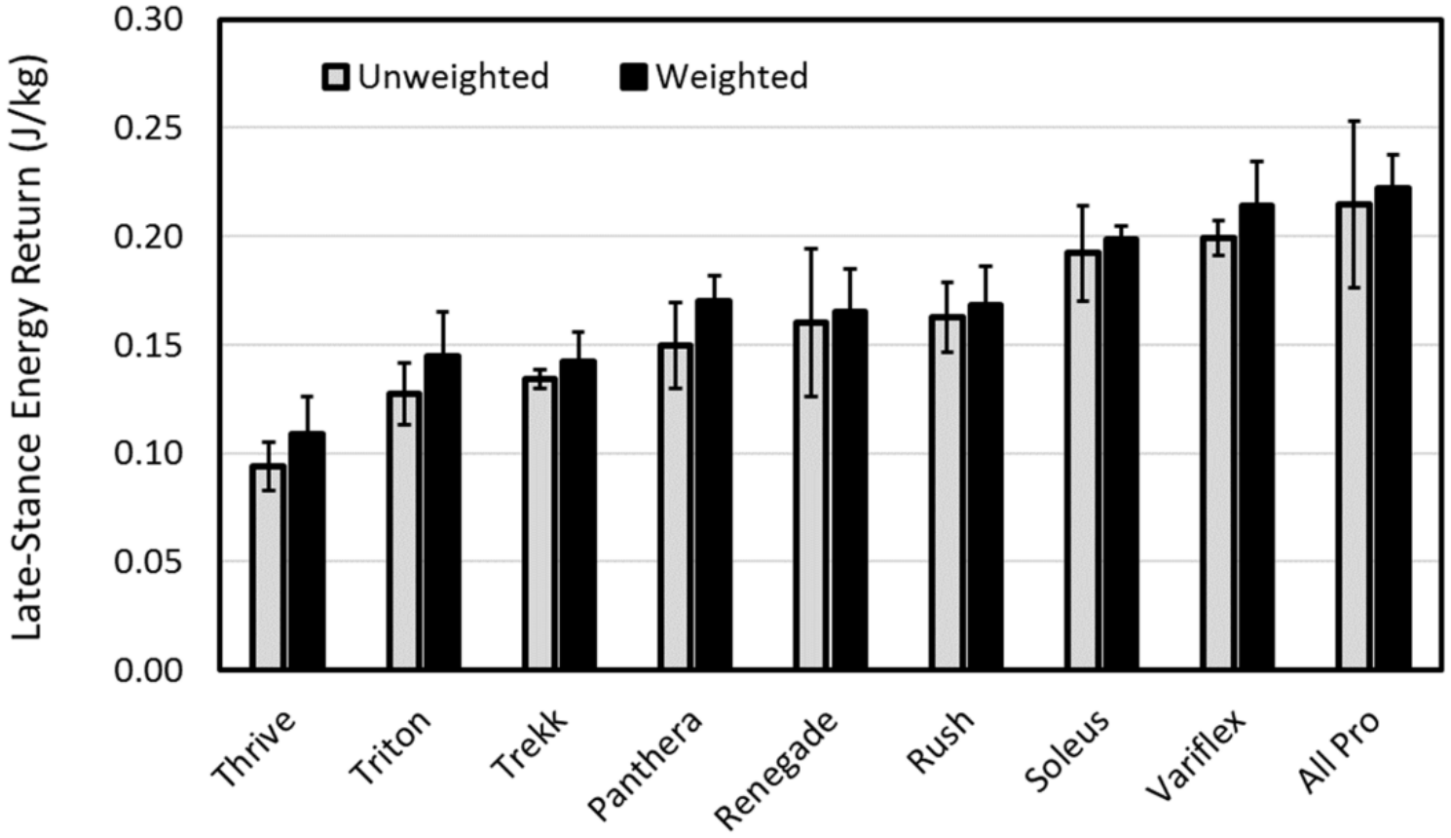
Mean (± 1 standard deviation) late-stance energy return across all subjects sorted from the least (left) to most (right) unweighted energy return.

Finally, Fig 10 shows the mean late-stance energy return normalized by mass (including the 22-kg mass when relevant) across all subjects for the unweighted and weighted walking conditions. For the unweighted walking condition, the mean late-stance energy return across feet was 0.159 ± 0.038 J/kg and ranged from 0.094 ± 0.011 J/kg for the Thrive to 0.215 ± 0.038 J/kg for the All Pro. For the weighted walking condition, the mean late-stance energy return across feet appeared to increase only slightly to 0.170 ± 0.036 J/kg and ranged from 0.109 ± 0.017 J/kg for the Thrive to 0.222 ± 0.015 J/kg for the All Pro.

## Discussion

While many Service members and Veterans with lower-limb amputation have the potential for high function, objective criteria for evaluating and prescribing appropriate ankle-foot prostheses is currently lacking. Specifically, for Service members with a lower-limb amputation, it is unclear which prosthetic ankle-foot systems best accommodate load carriage while also providing good overall function and mobility for unweighted activities. Compared to healthy, intact ankle-foot systems that adapt to added load by maintaining similar ankle motion and effective rocker shapes during walking [12], most prosthetic feet are spring-like and continue to bend with added load. Naturally, preventing this bending through the prescription of prosthetic feet with stiff forefoot keel structures would seem to best mimic the physiologic system that these devices are trying to replace. An important consideration in this prescription strategy, however, is that prosthetic feet with stiff forefoot keel structures are also less likely to provide sufficient late-stance energy return [14], possibly contributing to an increase in metabolic energy expenditure during physically demanding activities such as weighted walking. Understanding the relative tradeoff between roll-over shape invariance, changes in effective foot length ratio, and late-stance energy return as a function of forefoot keel stiffness is therefore an important consideration in prescribing prosthetic feet that meet the needs of high-functioning persons with lower-limb amputation. The goal of this study was to investigate the ability of currently available prosthetic ankle-foot systems to accommodate weighted walking by examining the mechanical characteristics (i.e., forefoot stiffness) and dynamic function (i.e., rocker radius, effective foot length ratio, late-stance energy return) of prosthetic feet designed for the highest activity users.

As expected, mechanical testing revealed that forefoot stiffness varied across all ankle-foot systems, both within foot type and between foot type. Variations within foot type are evident from the different order (left to right) of load versus deflection curves shown for each subject in Fig 4. These variations are likely due to the fact that the body weight and foot length of each subject was different, resulting in different foot sizes, spring categories, and therefore, slightly different mechanical properties within each foot type. Furthermore, the magnitude of loading used to calculate K_ABW_ was relative to the users’ body weight, potentially resulting in load-dependent variability within each foot type. Larger differences in mechanical designs between foot type resulted in larger variations in forefoot stiffness across feet. According to the results shown in Fig 5, the All Pro, Variflex, and Soleus feet appeared to provide the least forefoot stiffness at loads above body weight and the Triton, Trekk, and Thrive feet appeared to provide the most forefoot stiffness. In particular, the Thrive appeared to have the stiffest forefoot, likely owing to its unique design: a full-length primary keel that progressively comes into contact with a secondary, upper keel to provide additional support to added loads. Seemingly this design feature would best accommodate load carriage by resulting in smaller changes in roll-over shape radius and effective foot length ratio during weighted walking.

Contrary to our hypothesis, this result was not readily apparent in the subsequent analysis of ankle-foot roll-over shape data. Instead, while appreciable changes in forefoot stiffness were observed across all feet during mechanical testing, roll-over shape profiles appeared largely insensitive to the effects of load carriage. Fig 6 shows the effective roll-over shape of two feet on the extremes of forefoot stiffness—the All Pro (i.e., least stiff) and the Thrive (i.e., most stiff). Despite appreciable differences in stiffness profiles between these feet, both exhibited relatively small changes in roll-over shape radii during weighted walking (Fig 8). A possible explanation for this result may be that while wearing the All Pro, Subject B exhibited a smaller roll-over shape radius during unweighted (versus weighted) walking, resulting in a negative change in radius that decreased the overall mean (and increased the standard deviation) reported in Fig 8. However, even without this conflicting result, overall changes in roll-over shape radii, which ranged from 0.003 ± 0.025 (All Pro) to 0.028 ± 0.009 (Triton), correlated poorly with K_ABW_ (linear curve fit, r^2^ = 0.03) and were similar in magnitude to the approximate change (0.015) in roll-over shape radius observed in the ankle-foot system of an able-bodied population walking with a comparable 23-kg weighted vest [12]. Accordingly, changes observed in roll-over shape radii across prosthetic feet with different forefoot stiffness profiles all appeared to be within a physiological “normal” range.

The clinical implications of varying rocker radius and foot length on the energetic cost of walking have been investigated previously by Adamczyk and Kuo [20], who found that foot length (versus radius) has a much greater effect on both the mechanical work of the step-to-step transition and the overall energetic cost of walking. In this previous study, net metabolic rates were estimated from respiratory gas exchange data collected during treadmill trials while able-bodied subjects wore custom-made walking boots with interchangeable bottom surfaces designed with different foot radii and foot lengths. Five of these surfaces had a foot radius of 0.4 m with different foot lengths (0.203, 0.229, 0.254, 0.279, 0.305 m) and two of these surfaces had a foot length of 0.254 m with different foot radii (0.3 and 0.6 m). Within the range of radii tested (300 mm total), metabolic rate did not change significantly, suggesting that the mean changes observed across feet in the present study (0.003–0.028; equivalent to 6–48 mm) probably did not have a significant effect on the energetic cost of walking. Likewise, while varying foot length (within a 100-mm range) has been shown to more significantly affect walking energetics, the magnitude of changes observed in the present study (Fig 9; minimum = 0.026 ± 0.019; equivalent to 7 ± 5 mm with the Thrive; maximum = 0.053 ± 0.016; equivalent to 14 ± 4 mm with the All Pro) also appeared relatively small and therefore, did not likely affect walking energetics.

Beyond these studies, others have shown that reductions in effective foot length may also contribute to a drop-off effect that could lead to a shorter step length on the contralateral foot and a more forceful loading of the sound side limb during weighted walking activities [13,16,21]. For example, in a study of transtibial prosthesis users by Hansen et al. [16], simple modifications were used to alter the effective forefoot rocker length of a Shape&Roll prosthetic foot to 62%, 74%, and 82% of its total length. At both normal (1.0–1.2 m/s) and fast (1.4–1.6 m/s) walking speeds, a significant difference in the symmetry of the first peak of the vertical ground reaction force was found between the 74% and 82% foot length conditions, corresponding to a difference in effective foot length of approximately 8%. While the mean changes in effective foot length across all feet in the present study were less than 8%, additional studies are needed to confirm whether these reductions may in fact cause more forceful loading on the sound side, particularly at fast walking speeds, which were not investigated in the present study.

The apparent insensitivity of roll-over shape parameters to weighted walking in the present study suggests that a more important consideration in prescribing prosthetic feet for high activity users may instead be the effect of forefoot stiffness on late-stance energy return. Indeed, late-stance energy return appeared highly sensitive to forefoot stiffness, with the least stiff feet (All Pro, Variflex, Soleus) providing the most late-stance energy return and the stiffest feet (Thrive, Triton, Trekk) providing the least late-stance energy return. These results are also in agreement with those of a previous study by Fey et al. [14], which found that compliant feet tended to increase late-stance dorsiflexion, mid-stance energy storage, late-stance energy return, and intact and residual muscles activity, especially in the muscles responsible for body support. The authors of this previous study concluded that while foot compliance may be beneficial for prosthesis users with strong quadriceps and good control of these muscles, the net contribution to forward propulsion and swing initiation appears limited by the amount of additional muscle activity needed for body support. Furthermore, in a more recent study by the same group, a forward dynamic model was used to find that net metabolic cost was actually minimized when the nominal stiffness of the prosthetic toe and mid-foot was increased and the nominal stiffness of the heel and ankle was decreased [22]. Accordingly, forefoot stiffness clearly has an important effect on late-stance energy return, however the relationship between forefoot stiffness and net metabolic cost is influenced by the stiffness in other regions of the prosthetic forefoot as well as the strength of intact and residual-limb musculature that supports and propels the body forward during walking.

Collectively, these study results highlight several important paths for future investigation. In the current study, three users with different body weights and activity levels each walked with nine commercially available prosthetic feet, applying functionally relevant loading profiles to a total sample size of 27 different ankle-foot systems. This study design allowed for a thorough investigation of prosthetic feet designed for MFCL 4 users, guiding future clinical testing of these systems. However, to understand the statistical and clinical significance of changes observed in the rocker shape and late-stance energy return of these feet, future studies should include activities beyond that of level walking and involve a larger, more diverse study population. Using the roll-over shape radius data collected in this study, we ran a power analysis for a one-tailed paired t-test using G*Power 3.1. Effect size (d) was calculated using the mean and standard deviation difference in rocker radius between the unweighted and weighted conditions (across subjects) for one foot with the consistently lowest (Soleus) and one foot with the consistently highest (Thrive) forefoot stiffness according to Fig 4. Assuming a correlation between groups of 0.5 (resulting in an effect size d = 0.97), α = 0.05, and power = 90%, a sample size of 11 subjects would be needed in a future clinical study to determine a significant difference in radii of these two prosthetic ankle-foot systems.

Future studies should also consider the effect of weight distribution about the torso, walking speed, and prosthetic alignment on the dynamic characterization of prosthetic ankle-foot systems designed for high activity users. Indeed, several previous studies of weighted walking have used different weight distribution methods (e.g., backpacks) to simulate common scenarios of load carriage (e.g., [3,6,7]). While the results of these previous studies may not be entirely generalizable to the present study, the methodological decision to utilize a weighted vest in the present study was based on ecological validity, resulting in a protocol that more closely simulates the weight distribution of protective gear and weapons/ammunition carried during dismounted operations in the military. Furthermore, walking speed was controlled in the present study to isolate the effect of added weight on gait. It is possible, however, that subjects may have been forced to walk in a manner that was not optimal or preferred (e.g., subjects may have preferred a slower walking speed while carrying added weight). Finally, in the present study, alignment was clinically optimized for each foot by a certified prosthetist, following standard clinical procedures. Prior work has shown that this approach can reduce differences between feet, compared to an approach of keeping the alignment constant between feet [17]. We believe the approach adopted in the present study is more clinically relevant and allows the clinician to determine if there are still meaningful differences between feet after clinically optimized alignment.

With regard to mechanical testing, it is important to note that only one angle was used to test and analyze forefoot deflection, and that more comprehensive testing configurations, such as those outlined by ISO 22675, may provide additional insight into the overall dynamic response of prosthetic feet designed for high activity users. Furthermore, future analyses should consider the contribution of overall stiffness and vertical compliance on the assessment of prosthetic feet designed to accommodate heavy load carriage and to what extent heel stiffness affects weighted walking.

## Conclusions

According to the mechanical and human subject testing performed in this study, prosthetic feet with a range of forefoot stiffness profiles exhibited minimal changes in roll-over shape radii and effective foot length ratio measured during weighted walking compared to unweighted walking. At the same time, prosthetic feet with more compliant forefoot keel structures appeared to provide more late-stance energy return compared to feet with stiffer keels, both during the weighted and unweighted walking conditions. The results of this study may be useful in providing a guide for the prescription of prosthetic feet for high activity users. For examples, prosthetic feet that feel too soft or too stiff can be replaced with other foot types that are stiffer or more compliant according to the data presented in Fig 5. The results of this study also suggest that prosthetic ankle-foot systems with compliant forefoot keel structures may better accommodate weighted walking by reducing the metabolic cost of high-impact activities. However, other factors, such as the residual-limb strength of the user, the overall stiffness profile of the prosthetic foot, and the durability of the prosthesis in response to sudden impacts, should be considered in combination with these results to more fully understand the functional implications of prescribing prosthetic feet with different forefoot keel properties.

## Supporting information

S1 File Minimal data underlying study results. (XLSX)
